# Force and Its Transmutations

**Published:** 1877-11

**Authors:** 


### FORCE AND ITS TRANSMUTATIONS.

Modern science has shown conclusively that force is nowhere *produced*, however many and Protean may be the forms in which it appears. So far as our present knowledge extends, the amount of force in the universe is a fixed quantity, which can be neither increased nor diminished. In loose every-day language we speak of the gain of power by the use of machines, but in reality no such gain in possible. What seems like it is nothing but the exchange of power for its exact equivalent, an apparent gain in force being paid for by a proportionate loss in speed, or *vice versa.* If a pound lifts ten pounds it must move through tenfold the distance, or the account must be balanced in some other way. Machines are mere conveniences for the application of power, whether it be that of our own muscles or be drawn from the immense supply of force which nature places at our command. By their aid we compel the winds and the waters to labor for us, and make the fire and the lightning our servitors. The Arabian fable of the lamp of Aladdin becomes a familiar fact, and genii mightier and more beneficent than Oriental fancy ever dreamed of perform at our bidding miracles of strength and skill beyond the most extravagant feats recounted in the tales of Scheherezade.

When force seems to be produced it is only because it has been imprisoned, like the genii in the leaden casket, and is now set free. Thus water and coal are inert substances, and apparently incapable of exerting any force; but set the coal on fire and convert the water into steam, and we get an expansive power in the heated vapor which gives our engines their gigantic energies, concentrating the strength of a thousand men in one little piece of mechanism.

But where does the force come from in this case? Was it really imprisoned in the water or in the coal, as by the figure we might imply? Some one will say that it consists in the *heat* which gives the vapor its expansive energy. But whence does the heat come? You answer again that it is from the burning of the coal, which is nothing more than the chemical combination of the carbon with the oxygen of the air. Here a new force comes in, which we know as *affinity*, or chemical attraction. A mighty force it is, indeed, which draws the atoms of carbon and oxygen together with such inconceivable velocity that their collision gives rise to the heat, as iron grows hot under repeated blows of a hammer. Out of the clashing of countless particles infinitesimally minute, acted upon by this intense form of attraction, is born all that fervid force which is the life of the steam-engine.

What a tremendous source of power this affinity is we scarcely appreciate until we actually measure it. and find that the heat given out by a pound of coal in combining with oxygen is sufficient, when converted into mechanical motion, to raise more than a million pounds through a distance of one foot, or to hurl a ton nearly to the height of a mile. The burning of about three hundred pounds of coal represents the hard labor of an able-bodied man for a whole year.

In a certain sense, then, we, may say that the force was imprisoned in the coal. To obtain the force we must have carbon in an elementary state for the oxygen to combine with. After the diverse atoms have rushed together we can get no more heat from the affinity that unites them as carbonic acid,—unless, indeed, we can tear them from their hold upon each other, and let them clash again. But to separate them would evidently require a greater force than was exerted in their combination and now binds them together.

Where shall we find the agent potent enough to accomplish this divorce of the atoms after affinity has once united them? Not in the thunder nor in the whirlwind, not in any fierce and violent exercise of elemental forces, but in an agency that reminds us rather of the "still small voice" that announced to the waiting prophet the coming of the Almighty. The sunbeam falling on a leaf is the silent but irresistible power that undoes the work of affinity, and bids the burnt carbon rise phœnix-like from its ashes to resume its elementary form. Every leaf that flutters in the summer air and absorbs the sunbeams is a chemical laboratory in which this marvellous decomposition of carbonic acid is going on all day long, the oxygen being poured forth into the air and the carbon packed away in the tissues of the plant.

From this store-house it may eventually be taken and burned by man, serving him anew as a source of heat and force; and this series of transformations may be repeated again and again.

But where did the coal in the mine come from? From the leaf and the sunbeam, precisely like the wood we burn upon the hearth. Uncounted ages before man lived on this earth, a strange luxuriant vegetation flourished upon its surface, absorbing the carbonic acid and the sunlight as plants do to-day; and the coal-beds that supply us with fuel are the buried remains of that pre-Adamite growth. When we sit in winter before the coal fire glowing in the parlor grate, we are virtually basking in the sunlight that fell upon our planet millions upon millions of years before the creation of man. The light and heat of that sunshine were caught and imprisoned by the leaf, and have been locked up in the plant through its life and death and long entombment in the earth. The steam-engine is a modern machine, but the source of its power is incalculably more ancient than the origin of the human race. The fuel that heats its boilers was fabricated by the subtle fingers of the sunbeam in the delicate cells of the leaf when this world of ours was young and as yet unfitted for the abode of man. The engine is a piece of human design, the result of intellect working out a premeditated end. Was not the preparation of the coal a part of a Divine plan, the work of a Supreme Mind carrying out a benevolent purpose? Shall we believe that coal and mind and engine, are only steps in an "evolution" effected by the action of blind material forces, unguided by intellect and will; or shall see beyond and above those forces an infinite wisdom, power, and love, contriving, conducting, and inspiring all?

We have sketched but a single chapter, so to speak, in the history of the transmutation of force; and it is only a restatement of what was already known to many who may read it. But there are many others to whom these grand generalizations of modern science are not so familiar, and to whom this brief and simple presentation thereof may not be unacceptable or unprofitable. Indeed, they are wonderful enough to bear being oft repeated, even to those who are not ignorant of them.—*Boston Journal of Chemistry.*

